# The Activity of YCA1 Metacaspase Is Regulated by Reactive Sulfane Sulfur via Persulfidation in *Saccharomyces cerevisiae*

**DOI:** 10.3390/antiox13050589

**Published:** 2024-05-10

**Authors:** Qingda Wang, Xiaokun Zhang, Zhuang Du, Honglei Liu, Yongzhen Xia, Luying Xun, Huaiwei Liu

**Affiliations:** 1 State Key Laboratory of Microbial Technology, Shandong University, Qingdao 266237, China; wangqingda@sdu.edu.cn (Q.W.); 202212552@mail.sdu.edu.cn (X.Z.); 202020141136@mail.sdu.edu.cn (Z.D.); lhl@sdu.edu.cn (H.L.); xiayongzhen2002@sdu.edu.cn (Y.X.); luying_xun@vetmed.wsu.edu (L.X.); 2 Department of Chemistry, School of Molecular Biosciences, Washington State University, Pullman, WA 99164-4630, USA

**Keywords:** YCA1, metacaspase, reactive sulfane sulfur, persulfidation, chronological lifespan, apoptosis

## Abstract

YCA1, the only metacaspase in *Saccharomyces cerevisiae*, plays important roles in the regulation of chronological lifespan, apoptosis, and cytokinesis. YCA1 has protein hydrolase activity and functions by cleaving itself and target proteins. However, there are few reports about the regulation of YCA1 activity. In this study, we observed that reactive sulfane sulfur (RSS) can inhibit the activity of YCA1. In vitro experiments demonstrated that RSS reacted with the Cys_276_ of YCA1, the residue central to its protein hydrolase activity, to form a persulfidation modification (protein-SSH). This modification inhibited both its self-cleavage and the cleavage of its substrate protein, BIR1. To investigate further, we constructed a low-endogenous-RSS mutant of *S. cerevisiae*, BY4742 Δ*cys3*, in which the RSS-producing enzyme cystathionine-γ-lyase (CYS3) was knocked out. The activity of YCA1 was significantly increased by the deletion of CYS3. Moreover, increased YCA1 activity led to reduced chronological lifespan (CLS) and CLS-driven apoptosis. This study unveils the first endogenous factor that regulates YCA1 activity, introduces a novel mechanism of how yeast cells regulate chronological lifespan, and broadens our understanding of the multifaceted roles played by RSS.

## 1. Introduction

Cell aging is a process in which a cell’s proliferation, differentiation, and other physiological functions gradually decline over time. It occurs throughout the entire lifespan and is crucial to embryonic development [1], tissue repair [2], and tumor suppression [3]. *S. cerevisiae* is a eukaryotic model organism widely utilized in the study of cell aging [4,5,6]. There are two types of cell aging in *S. cerevisiae*, chronological aging and replicative aging, which can be monitored by using chronological lifespan (CLS) and replicative lifespan (RLS), respectively. CLS represents the survival of a cellular population in the postmitotic, nondividing phase. RLS represents the number of cell divisions a mother cell can undergo before death. *S. cerevisiae* also possesses functional and highly conserved apoptotic machinery. Recent studies have indicated a close relationship between the aging process and apoptosis in yeast [7,8,9].

YCA1, the only metacaspase in *S. cerevisiae*, plays a role in regulating the apoptosis induced by aging [10,11]. YCA1 is a homolog of caspases and possesses proteolytic activity. The activation of YCA1 occurs through self-cleavage, and the fragment containing the active cysteine can cleave target proteins in a manner similar to caspases. It has been observed that overexpressing YCA1 can enhance apoptosis in yeast cells [12]. Furthermore, aging cells exhibit increased presence of activated YCA1 fragments [13]. Therefore, the proteolytic activity of YCA1 appears to be crucial to its regulation function in aging-related apoptosis.

In recently years, reactive sulfane sulfur (RSS) has been discovered in both eukaryotic and prokaryotic cells [14,15], including hydrogen polysulfide (HS_n_H, n ≥ 2), organic polysulfide (RS_n_H, n ≥ 2), and polysulfane (RS_n_R, n ≥ 2). In *S. cerevisiae*, endogenous RSS is mainly produced by cystathionine γ-lyase (CSE), cystathionine β-synthase (CBS), Cysteinyl-tRNA synthetase 2 (CRS2), and rhodanese (Rhod). The highest amount of endogenous RSS reaches about 400 μM [16,17,18,19]. Previous studies have reported that H_2_S can stimulate yeast cells and lead to extended cell lifespan [20,21]. However, more recent studies indicate that the effects of H_2_S are actually attributed to RSS [22,23]. RSS can directly react with cysteine residues of proteins to form protein-SSH, a process now called protein persulfidation or sulfhydration. This post-translational modification can alter protein activities. So far, a number of proteins have been reported to undergo such modification, such as caspase 3, lactate dehydrogenase A, and pyruvate kinase [24,25,26]. These proteins play important roles in cell metabolism, cell proliferation, and apoptosis processes.

In this study, we found that RSS was able to mediate the persulfidation of YCA1. Persulfidated YCA1 inhibited its self-cleavage activity, and the activity of cleaving target proteins was also lost. We knocked out cystathionine γ-lyase to reduce intracellular RSS in *S. cerevisiae* and found that the proteolytic activity of YCA1 was promoted. Moreover, reducing intracellular RSS also promoted apoptosis and reduced cell lifespan, two processes in which YCA1 is closely involved. This study revealed a new post-translational modification that occurs in YCA1 which plays important role in regulating YCA1 activity, thereby affecting cell aging.

## 2. Materials and Methods

### 2.1. Strains and Materials

*S. cerevisiae* BY4742 *(MATα his3*Δ*1 leu2*Δ*0 lys2*Δ*0 ura3*Δ*0*) and its mutants were cultured in yeast extract–peptone–dextrose (YPD) medium at 30 °C. The constructed mutants and plasmids are listed in Supplementary Materials (Table S1). *Escherichia coli* DH5α and BL21(DE3) strains were cultured in lysogeny broth (LB) medium at 37 °C. Calcium chloride and sodium hydrosulfide (NaHS) were purchased from Sigma-Aldrich (Saint Louis, MO, USA). The Annexin V-FITC/PI Cell Apoptosis Detection Kit was purchased from TransGen Biotech (Beijing, China). HS_n_H were prepared following the protocol of a previous study [27]. Other chemicals were purchased from local companies if not specifically mentioned.

### 2.2. S. cerevisiae BY4742 Mutant Construction

For the construction of BY4742 Δ*cys3*, *cys3* was deleted by using the short flanking homology (SFH) method based on the KanMX4 deletion cassette [28]. The primers were designed to amplify the loxP-KanMX4-loxP cassette from the plasmid pUG6. The forward primer had a 50-nucleotide extension corresponding to the region upstream of *cys3*, while the reverse primer had a 50-nucleotide extension corresponding to the region downstream of *cys3*. The obtained PCR fragments were used to transform the BY4742 strain by using the lithium acetate procedure [29]. Transformed cells were selected by YPD solid medium containing 200 mg/L Geneticin (G418). For the construction of BY4742 Δ*cys3* Δ*yca1*, *yca1* was deleted in the obtained Δ*cys3* mutant. Primers were designed to amplify the loxP-His3MX6-loxP cassette from the plasmid pFA6a-GFP(S65T)-His3MX6. The forward primer had a 50-nucleotide extension corresponding to the region upstream of *yca1*, while the reverse primer had a 50-nucleotide extension corresponding to the region downstream of *yca1*. The gene deletion processes were the same, except that the mutant cells were selected on synthetic dropout (SD) medium.

### 2.3. Protein Expression and Purification

The gene encoding YCA1 was amplified from the genomic DNA of *S. cerevisiae* BY4742. The gene encoding the N-terminal fragment of BIR1 was codon-optimized and chemically synthesized. The YCA1-encoding gene was ligated with the pET21b plasmid by using the T5 exonuclease-dependent assembly method [30]. The BIR1-encoding gene was ligated to the pET15b plasmid by using the same method. For protein expression and purification, *E. coli* BL21 (DE3) strains harboring the expression plasmids were incubated in LB medium at 30 °C with shaking (225 rpm). Kanamycin (50 μg/mL) was added. When OD_600_ reached 0.6, 0.1 mM isopropyl β-D-1-thiogalactopyranoside (IPTG) was added to induce expression, and the temperature was decreased to 16 °C. After cultivation for an additional 24 h, cells were harvested by centrifugation and re-suspended in buffer I (20 mM Tris-HCl, 0.5 M NaCl, and 20 mM imidazole (pH 8.0)). Cell disruption was performed by using a Pressure Cell Homogeniser (SPCH-18) at 4 °C. The obtained cell lysate was centrifuged to eliminate debris. Target proteins in the supernatant were initially purified by using nickel-nitrilotriacetate (Ni-NTA) agarose. The obtained proteins were then subjected to further purification by passing them through a size exclusion column.

### 2.4. Analysis of Inhibitory Effect of RSS on YCA1 Cleavage Activity

To test the self-cleaving activity of YCA1, 5 μM purified YCA1 was added to 200 μL of reaction buffer (20 mM Tris-HCl (pH 8.0) and 150 mM NaCl) containing different concentrations of HS_n_H (0–150 μM) and then incubated at 37 °C for 30 min. Subsequently, 10 mM CaCl_2_ was added, and the reaction system was further incubated at 18 °C for 12 h. The self-cleavage of YCA1 was analyzed by SDS-PAGE. To test the activity of YCA1 of cleaving BIR1, 5 μM YCA1 was added to 200 μL of reaction buffer (20 mM Tris-HCl (pH 8.0) and 150 mM NaCl) containing different concentrations of HS_n_H (0–150 μM) and then incubated at 37 °C for 30 min. Subsequently, 5 μM BIR1 and 10 mM CaCl_2_ were added, and the reaction system was incubated at 18 °C for 12 h. The fragments of cleaved BIR1 were analyzed by SDS-PAGE.

### 2.5. Protein LC-MS/MS Analysis

The purified YCA1 (4.5 mg/mL) was mixed with 200 μM HS_n_H or DTT in Tris-HCl buffer (50 mM; pH 7.4). After incubation at 25 °C for 30 min, the mixture was loaded onto a PD-10 desalting column to remove unreacted HS_n_H or DTT. The obtained protein sample was treated with iodoacetamide (IAM) and subsequently digested with trypsin, following a previously established protocol [31]. The analysis was performed by using the Prominence nano-LC system (Shimadzu, Shanghai, China) equipped with a custom-made silica column (75 μm × 15 cm) packed with 3 μm Reprosil-Pur 120 C18-AQ. The elution process involved a 100 min gradient ranging from 0% to 100% of solvent B (0.1% formic acid in 98% acetonitrile) at a flow rate of 300 nL/min. Solvent A was composed of 0.1% formic acid in 2% acetonitrile. The eluent was ionized and electrosprayed via the LTQ-Orbitrap Velos Pro CID mass spectrometer (Thermo Scientific, Shanghai, China), which operated in data-dependent acquisition mode using Xcalibur 2.2.0 software (Thermo Scientific, Shanghai, China). Full-scan MS spectra (ranging from 400 to 1800 *m*/*z*) were detected with the Orbitrap at a resolution of 60,000 at 400 *m*/*z*.

### 2.6. Analysis of Growth Curve

*S. cerevisiae* BY4742 strains were inoculated in 5 mL of YPD medium and cultured at 30 °C under shaking (200 rpm) for 12 h. The culture was centrifuged at 3000× *g* for 5 min, and the obtained cells were resuspended in YPD medium (OD_600_ = 0.1). The cells were then incubated at 30 °C, and their growth was assessed by measuring OD_600nm_ by using a spectrophotometer.

### 2.7. Analysis of Intracellular RSS Level

Intracellular RSS levels of *S. cerevisiae* BY4742 cells were assessed by using a previously reported method [32]. In brief, BY4742 cells were suspended in 100 µL of reaction buffer (50 mM Tris-HCl and 1 mM sulfite (pH 7.5)) and incubated at 95 °C for 30 min. During incubation, sulfane sulfur atoms reacted with sulfite to form thiosulfate (S_2_O_3_^2−^). Monobromobimane (mBBr) was then used to derivatize the formed thiolsulfate, and the derivative was quantified by using HPLC (LC-20A; Shimadzu, Kyoto, Japan) with a fluorescence detector (RF20A; Shimadzu, Kyoto, Japan). The obtained S_2_O_3_^2−^ derivative concentration represented the intracellular RSS level.

### 2.8. Analysis of YCA1 Activity In Vivo

Green fluorescent protein (GFP) was fused to the C-terminus of BIR1 in the BY4742 wt and Δ*cys3* chromosome by using the one-step PCR-mediated gene disruption method. The primers were designed to amplify the GFP (S65T)-His3MX6 cassette from the plasmid pFA6a-GFP(S65T)-His3MX6. The forward primer had a 50-nucleotide extension corresponding to the region upstream of the BIR1 C-terminus, while the reverse primer had a 50-nucleotide extension corresponding to the region downstream of the BIR1 C-terminus. Recombinant strains were selected on SD medium. The recombinant BY4742 wt and Δ*cys3* strains were cultivated to stationary phase in YPD medium; then, cells were collected and disrupted to extract total protein. The total protein concentration was measured by using the BCA method and adjusted to 5 mg/mL. The cleavage of BIR1-GFP by YCA1 was assessed by using Western blot. A polyclonal antibody targeting GFP was used.

### 2.9. Chronological Lifespan Assay

Chronological lifespan was analyzed by using the method reported previously [33]. Briefly, *S. cerevisiae* BY4742 strains were cultured in 5 mL of SD medium under shaking (200 rpm) at 30 °C overnight. Then, cultures were diluted to an initial density of 10^6^ cells/mL (OD_600_ of 0.1) in 100 mL of SD liquid medium. Cultures were incubated in flasks with a volume/medium ratio of 5:1 at 30 °C under shaking (220 rpm). After 3 days, survival rate monitoring was initiated by measuring the ability of individual yeast cells/organisms to form colonies (colony-forming units [CFUs]). Specifically, cultures were serially diluted to achieve a 1:10,000 dilution in sterile distilled water, followed by plating 100 µL of this dilution onto YPD agar medium. The number of CFUs on day 3 was deemed as the initial CFU.

### 2.10. CLS-Driven Apoptosis Assay

*S. cerevisiae* BY4742 cells were cultured in 5 mL of SD medium and incubated at 30 °C under shaking (200 rpm) overnight. The cells were subsequently centrifuged at 3000× *g* for 5 min and resuspended in SD medium with an OD_600_ of 0.1. After 72 h of cultivation, cells were collected by centrifugation at 3000× *g*, and the cell number was adjusted to 1 × 10^6^.

The cells were washed twice with sorbitol buffer (1.4 M sorbitol, 40 mM HEPES, and 1.5 mM MgCl_2_ (pH 6.5)). Subsequently, the yeast cells were gently agitated in sorbitol buffer containing 10 mg/mL of lysing enzyme and then incubated at 30 °C for 1~3 h to prepare protoplasts. The obtained protoplasts were washed with 200 μL of Annexin V-FITC binding buffer and then resuspend in Annexin V-FITC binding buffer containing 5 μL of Annexin V-FITC and 10 μL of PI. The staining reaction was performed at room temperature for 20 min in the dark. Finally, stained protoplasts were analyzed with a flow cytometer (Accuri™ C6, BD Biosciences, Franklin Lakes, NJ, USA). For each sample, 20,000 protoplasts were analyzed.

### 2.11. Transcriptomic Analysis

*S. cerevisiae* BY4742 cells were cultured in 5 mL of YPD medium and incubated overnight at 30 °C under shaking (200 rpm). The cells were subsequently centrifuged at 3000× *g* for 5 min and resuspended in YPD medium with an OD_600_ of 0.1. The cells were cultivated until they reached the logarithmic phase (OD_600_ = 1.5). Cells were then harvested for transcriptomics analysis, which was performed at Shanghai Applied Protein Technology Co., Ltd. (Shanghai, China).

Total RNA was extracted. The enrichment of mRNA was carried out by using magnetic beads with Oligo (dT), followed by addition of a fragmentation buffer to randomly interrupt the mRNA. The first strand of cDNA was generated by using six-base random primers, and the second strand was synthesized by introducing a buffer, dNTPs, and DNA polymerase I. AMPure XP was employed to purify the double-stranded cDNA, and subsequent steps involved A-tailing and the connection of sequencing adapters. Fragment size selection was conducted by using AMPure XP beads, and the final cDNA library was obtained through PCR enrichment. The library was sequenced by using the Illumina NovaSeq 6000 platform. Clean data were obtained by eliminating reads containing adapters, poly-N, and low-quality sequences from the raw data. The clean reads were aligned with the *S. cerevisiae* BY4742 genome by using HISAT2. The FPKM value of each gene’s expression in each sample was calculated by using featureCounts software (2.0.4). Genes with a *p*-value < 0.05 and fold change > 2 were considered significantly differentially expressed.

## 3. Results

### 3.1. RSS Inhibited Proteolytic Activity of YCA1 In Vitro

**Figure 1:**
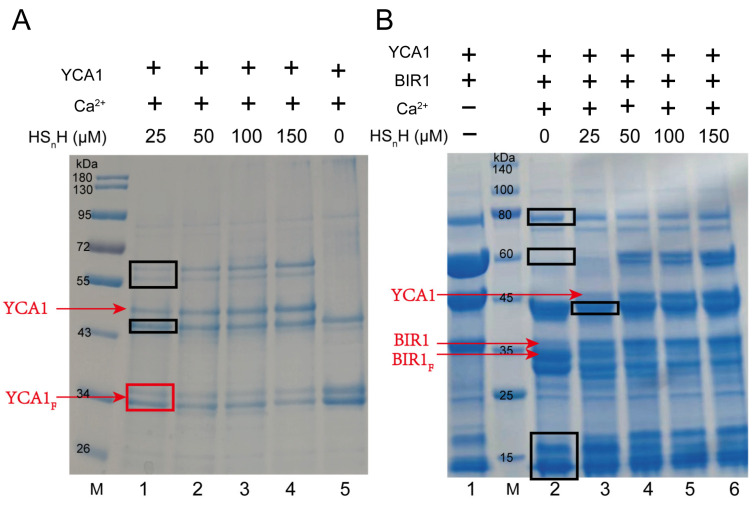
RSS inhibited self-cleavage and proteolytic activity of YCA1. (**A**) YCA1 at 5 μM was added to the reaction buffer. CaCl_2_ was added at a concentration of 10 mM. Different concentrations of HS_n_H were added (Lanes 1–4, 25 μM–150 μM). Lane 5 (no HS_n_H addition) was the control. Uncleaved YCA1 and cleaved YCA1 are labeled with red lines. YCA1_F_ represents cleaved YCA1. M represents the protein marker. (**B**) Purified YCA1 at 5 μM and BIR1 at 5 μM were mixed in the reaction buffer. Lane 1 contains only YCA1 and BIR1. Lane 2 contains YCA1, BIR1, and Ca^2+^ but no HS_n_H. CaCl_2_ (10 mM) was added in lanes 3~6. HS_n_H at 25 μM–150 μM were added in lanes 3~6. BIR1_F_ represents cleaved BIR1. M represents the protein marker. Contaminating proteins co-purified with YCA1 from *E. coli* are labelled with black boxes.

Freshly translated YCA1 has no proteolytic activity. It needs to undergo a self-cleavage process to activate such activity. Self-cleavage generates two fragments from the original YCA1, with the molecular weight of both being around 36 kDa. Only the C-terminal fragment shows proteolytic activity toward target proteins. The self-cleavage activity of YCA1 is dependent on the presence of Ca^2+^ [34]. When Ca^2+^ is not present, YCA1 is incapable of performing self-cleavage. We cloned the *yca1* gene from the *S. cerevisiae* BY4742 genome and expressed it in *E. coli* BL21 (DE3). The expressed protein was purified by using a nickel column. SDS-PAGE analysis demonstrated that the purified YCA1 thoroughly underwent self-cleavage in the presence of Ca^2+^ (Figure 1A). However, when HS_n_H was added into the YCA1 solution, self-cleavage was significantly inhibited, and the inhibition effect was more obvious at high concentrations of HS_n_H. Since HS_n_H solution inevitably contained a small portion of H_2_S (~3%; prepared by mixing S_8_ with H_2_S). It is possible that H_2_S chelates Ca^2+^ in the form of CaS, thereby inhibiting YCA1 self-cleavage. In our experiments, we used 10 mM Ca^2+^ and 25 μM~150 μM HS_n_H. We also mixed those amounts of Ca^2+^ and HS_n_H without YCA1 and found that no precipitate was produced. Therefore, the chelation reaction had no influence under our experimental conditions.

BIR1 (baculovirus IAP repeat 1) is one of the inhibitor-of-apoptosis (IAP) proteins. It was reported that BIR1 can be cleaved by active YCA1 [34]. We also cloned the gene encoding the N-terminal fragments of BIR1 (1-251aa) from the *S. cerevisiae* BY4742 genome and expressed it in *E. coli* BL21 (DE3). We used the purified BIR1 to assess YCA1 activity. BIR1 was mixed with YCA1 in the presence of Ca^2+^. SDS-PAGE analysis was performed to assess the cleavage. A smaller fragment appeared after BIR1 was cleaved (Figure 1B), which is consistent with the results reported previously [34]. When HS_n_H was added at concentrations ranging from 25 μM to 150 μM, the smaller fragment gradually disappeared, indicating that the proteolytic activity of YCA1 was inhibited.

### 3.2. Cys_276_ of YCA1 Was Reversibly Modified by RSS

**Figure 2:**
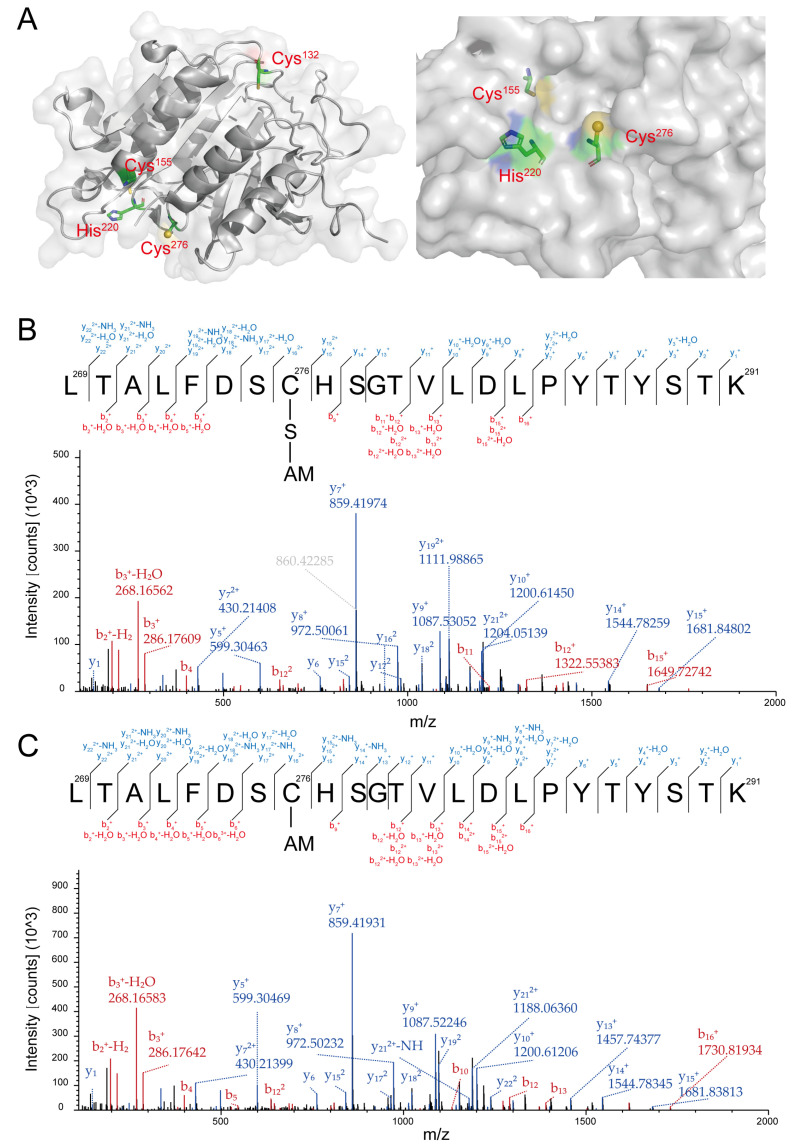
YCA1 was modified by RSS at the Cys_276_ position. (**A**) The crystal structure of YCA1. The data were obtained from PDB ID: 4f6o and were processed with PyMOL (2.0.7). (**B**) The MS^2^ spectra of the Cys_276_-containing peptide from the HSnH-reacted YCA1. “C-S-AM” represents the thiol group of Cys_276_ (Cys-SH) modified by persulfidation to form Cys-SSH, which is then blocked by IAM to form Cys-SS-AM. (**C**) The MS^2^ spectra of the Cys_276_-containing peptide from the DTT-treated YCA1. “C-AM” represents the thiol group of Cys_276_ (Cys-SH) blocked by IAM to form Cys-S-AM. The peptide fragments generated by the y, b ions are labeled in blue and red, respectively.

YCA1 contains three cysteines, Cys_132_, Cys_155_, and Cys_276_. According to its 3D structure determined by X-ray crystallography, Cys_276_ and His_220_ collectively form an active center. Unlike Cys_132_ and Cys_155_, Cys_276_ is located on the surface of YCA1, making it more susceptible to reacting with small compounds compared with the other two cysteine residues (Figure 2A; PDB ID: 4f6o).

RSS can affect protein activity via persulfidation modification. To examine whether HS_n_H inhibit the proteolytic activity of YCA1 also via this mechanism, we analyzed the HS_n_H-reacted YCA1 by using LC-MS/MS. A peptide containing Cys_276_ was detected with a molecular weight of 2621.22 Da (Figure S1A), corresponding to the Cys_276_-SSH modification (Figure 2B). No such modification was detected in the peptides containing Cys_132_ or Cys_155_. To investigate whether this modification was reversible, we treated the HS_n_H-reacted YCA1 with DTT and then analyzed it with LC-MS/MS. The Cys_276_-SSH modification disappeared in the same peptide (Figure 2C), as evidenced by a smaller molecular weight of 2589.25 Da (-32, corresponding to the loss of a sulfur atom) (Figure S1B). These results demonstrate that RSS reacted with the Cys_276_ residue of YCA1 to form persulfidation and that this modification was reversible.

### 3.3. RSS Inhibited Proteolytic Activity of YCA1 In Vivo

**Figure 3:**
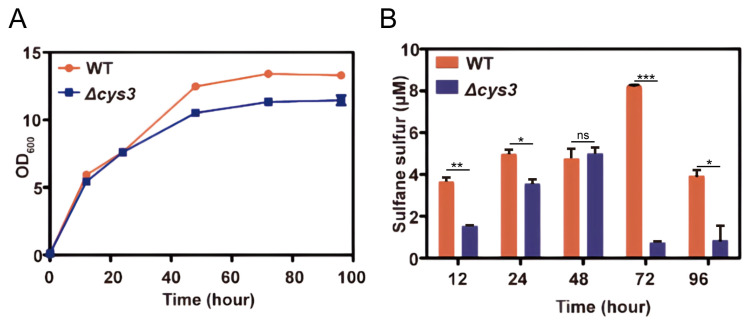
Knocking out *cys3* reduces intracellular RSS levels. (**A**) The BY4742 wt and Δ*cys3* strains were cultivated in YPD liquid medium. (**B**) The intracellular RSS levels of the BY4742 wt and Δ*cys3* at different times when cultivated in YPD medium. “*” represents a difference (*p* < 0.05), “**” represents a significant difference (*p* < 0.01), “***” represents a significant difference (*p* < 0.005) and “ns” represents no significant differences (*p* > 0.05) in the two-sided *t*-test. The data are from three replicated experiments and are shown as averages ± s.d.

The above experiments verified that RSS can affect YCA1 activity in vitro. However, the intracellular environment is much more complicated. To investigate whether the activity of YCA1 can be influenced by endogenous RSS in vivo, we first constructed an intracellular RSS level-altered strain. In *S. cerevisiae*, the *cys3* gene encodes the RSS-producing enzyme cystathionine γ-lyase. We knocked it out in *S. cerevisiae* BY4742. Compared with the wild type strain (wt), the mutant strain Δ*cys3* displayed slightly dampened growth when cultured in YPD medium (Figure 3A). Its intracellular RSS levels were obviously lower than those of the wt, especially in the stationary phase (72~96 h; Figure 3B).

**Figure 4:**
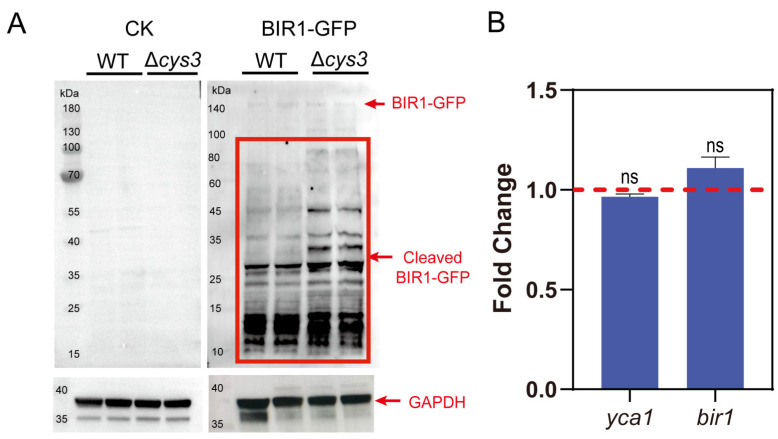
The activity of YCA1 of cleaving BIR1 was inhibited in Δ*cys3.* (**A**) The Western blot analysis of the intracellular fragments of BIR1-GFP. The BIR1-GFP cleaved fragments are indicated with red lines. “CK” represents BY4742 cells without BIR1-GFP. (**B**) Changes in the transcriptional levels of YCA1 and BIR1 in Δ*cys3*, with the wt as the control. The amounts of gene transcripts were from the transcriptomic analysis. The fold change was calculated by dividing the transcript amount in Δ*cys3* by that in the wt. “ns” represents no differences (*p* > 0.05) in the two-sided *t*-test. The data in (**B**) are from three replicated experiments and are shown as averages ± s.d.

Secondly, we constructed a BIR1-GFP fusion protein and expressed it in both the *S. cerevisiae* BY4742 wt and Δ*cys3* strains. To examine whether BIR1-GFP is cleaved by YCA1 in vivo, we conducted Western blot analysis by using the GFP antibody. Both uncleaved BIR1-GFP and its cleaved fragments were detected in both strains. However, the cleaved fragments were more abundant in Δ*cys3* than in the wt (Figure 4A), suggesting that the proteolytic activity of YCA1 was higher in Δ*cys3* than in the wt. BY4742 cells without BIR1-GFP were used as controls. Transcript analysis indicated that the transcription levels of *bir1* and *yca1* showed no significant changes in Δ*cys3* compared with the wt control (Figure 4B). Therefore, the increase in BIR1-GFP fragments in Δ*cys3* was not attributed to changes in the expression levels of these two proteins, but rather to the heightened proteolytic activity of YCA1, likely stemming from the reduced RSS level in Δ*cys3*.

### 3.4. Increased Proteolytic Activity of YCA1 Led to Short CLS and Early Apoptosis

**Figure 5:**
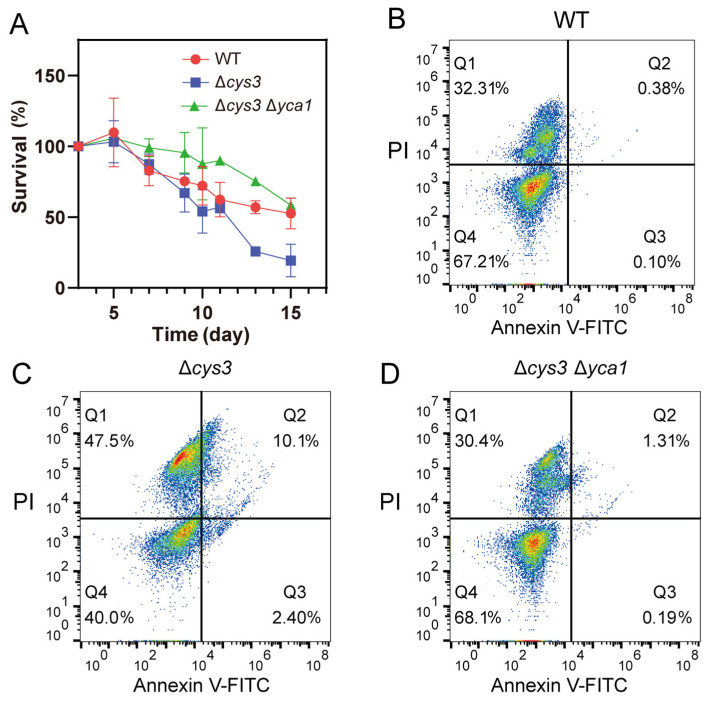
Increasing YCA1 activity resulted in physiological changes in *S. cerevisiae.* (**A**) The CLS of Δ*cys* was shorter than that of the wt and Δ*cys3*Δ*yca1*. (**B**–**D**) The apoptosis status of BY4742 wt, Δ*cys3*, and Δ*cys3*Δ*yca1* cells was analyzed by V-FITC/PI double staining after cultivation in SD medium for 72 h. The data in (**A**) are from three replicated experiments and are shown as averages ± s.d. The cell numbers are labeled in different colors, with red representing the highest cell count.

Previous studies indicated that activated YCA1 advances the CLS-driven apoptosis [12,13,35]. Given the increased proteolytic activity of YCA1 observed in Δ*cys3*, we wondered whether the CLS of Δ*cys3* cells was affected or not. A CLS analysis experiment was performed, revealing that the survival rate of Δ*cys3* cells rapidly declined to less than 30% after 12 days in SD liquid medium (Figure 5A). In comparison, the survival rate of wt cells remained above 50% even after 15 days. These results demonstrate that the CLS of Δ*cys3* was shorter than that of the wt. To confirm that the survival rate reduction was due to increased YCA1 activity, we constructed a Δ*cys3*Δ*yca1* mutant in which YCA1 activity was completely abolished. The survival rate of mutant cells was obviously higher than that of the wt on day 1~day 14 and became comparable to that of the wt on day 15. These findings collectively suggest that YCA1 has a reduction effect on CLS. Increasing YCA1 activity by reducing endogenous RSS can further enhance this effect.

To examine the apoptosis status of *S. cerevisiae* BW4742 cells after 72 h of cultivation, we used the apoptosis detection kit Annexin V-FITC and PI for staining. The stained cells were analyzed by using a flow cytometer. Log-phase yeast cells and yeast cells killed at high temperature were utilized to establish the boundaries of the four quadrants (Figure S2). For wt cells, we found that 67.21% of them were still vivid (Annexin V-FITC-, PI-; bottom left quadrant in Figure 5B; Q4), 32.31% were dead (Annexin V-FITC+, PI+; top right quadrant in Figure 5B; Q1), 0.38% were in the late apoptosis stage (Annexin V-FITC-, PI+; top left quadrant in Figure 5B; Q2), and 0.10% were in the early apoptosis stage (Annexin V-FITC+, PI-; bottom right quadrant in Figure 5B; Q3). For Δ*cys3* cells, we found that only 40.0% of them were vivid; 47.5% were dead; and 10.1% and 2.40% were in the late and early apoptosis stages, respectively. For Δ*cys3*Δ*yca1* cells, we found that 68.1% of them were vivid; 30.4% were dead; and 1.31% and 0.19% were in the late and early apoptosis stages, respectively. These results demonstrate that Δ*cys3* cells initiated the apoptosis processes much earlier than wt cells. Knocking out YCA1 significantly reduced the number of apoptotic and dead cells. These results indicate that increasing YCA1 activity by reducing endogenous RSS level led to early and severe apoptosis.

### 3.5. Systematic Analysis of Gene Expression Changes in YCA1-Activated Strain

**Figure 6:**
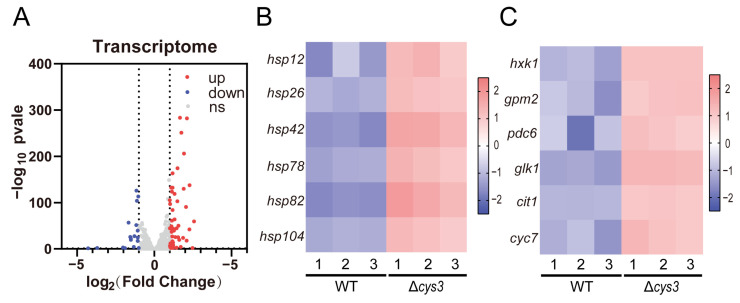
The transcriptomics analysis of the BY4742 wt and Δ*cys3* strains. (**A**) The number of genes changed significantly at the transcriptional level. (**B**) Heat shock protein genes were significantly up-regulated in Δ*cys3*. (**C**) Carbon metabolism-related genes were significantly up-regulated in Δ*cys3*. For each strain, three samples were collected and analyzed in parallel.

Transcriptomics analysis was conducted to systemically investigate the changes caused by increased YCA1 activity in Δ*cys3*. At the transcriptional level, 59 genes were up-regulated, and 18 genes were down-regulated (fold change > 2, *p* < 0.05) (Figure 6A and Table S2). Among the up-regulated genes, six heat shock protein-encoding genes (*hsp12*, *hsp26*, *hsp42*, *hsp78*, *hsp82*, and *hsp104*), which are associated with protein folding and the regulation of cellular chronological lifespan, were identified (Figure 6B). This finding is consistent with the observed reduced lifespan in Δ*cys3.* Additionally, four genes (*hxk1*, *gpm2*, *pdc6*, and *glk1*) involved in glycolysis and two genes (*cit1* and *cyc7*) involved in the tricarboxylic acid cycle were also up-regulated (Figure 6C), which is consistent with the impaired growth phenotype of Δ*cys3*.

## 4. Discussion

The YCA1 metacaspase plays important roles in regulating cell aging and apoptosis, primarily through its proteolytic activity. However, whether and how this activity is regulated by intracellular factors has remained poorly understood. In this study, we discovered that RSS, a family of sulfane sulfur species commonly present in all living cells, can influence the proteolytic activity of YCA1. In vitro experiments indicated that RSS directly reacted with YCA1, generating a persulfide bond in its Cys_276_ residue, which is an essential site for proteolytic activity [34]. This post-translational modification inhibited its self-cleavage, thereby reducing its capability of cleaving the target protein, BIR1. Further, we verified that reducing RSS content in *S. cerevisiae* cells also led to increased proteolytic activity of YCA1 in vivo. This finding is the first report that intracellular components can regulate YCA1 activity, opening up a new direction for studying the complex functions of YCA1.

In addition to the role of regulating cellular chronological lifespan, YCA1 is also involved in other crucial cellular processes, such as mitosis and the clearance of misfolded proteins. However, the specific downstream protein substrates targeted by YCA1 remain unclear. Transcriptomic analysis revealed that intracellular heat shock proteins were up-regulated, suggesting that the expression of these genes may be regulated by YCA1. Except for these functionally closely related genes, a number of genes related to carbon metabolism, sulfate assimilation, and amino acid synthesis were also significantly activated (Table S2). It is hard to say whether these changes were due to decreased YCA1 activity or *cys3* deletion. In addition, our data do not exclude the possibility that CYS3 and YCA1 act independently in the regulation of lifespan. Further studies are required for further clarification. Nonetheless, these results provide valuable insights into YCA1 research.

To summarize, cellular lifespan is typically intricately controlled. Herein, we reported that a biologically potent small molecule known as reactive sulfane sulfur can serve as a key regulator of YCA1 and cellular lifespan. Specifically, we recognized that RSS exerts its regulatory function by inhibiting the activity of YCA1 through persulfidation modification, thereby impeding the cleavage of its substrate proteins. These findings unveil the first endogenous factor that regulates YCA1 activity, introduce a novel mechanism of how cells modulate chronological lifespan, and broaden our understanding of the multifaceted roles played by intracellular RSS.

## 5. Conclusions

YCA1 is the only metacaspase in *S. cerevisiae* responsible for regulating chronological lifespan. Herein, we discovered that the intracellular small-molecule reactive sulfane sulfur is capable of regulating YCA1 activity. RSS modified the active cysteine of YCA1 by persulfidation, thereby inhibiting its self-cleavage and the cleavage of the substrate proteins. Yeast cells exhibited reduced chronotropic lifespan and increased CLS-driven apoptosis due to inhibition of YCA1 activity by reducing the content of intracellular reactive sulfane sulfur. Thus, reactive sulfane sulfur plays important roles in regulating the chronological lifespan of yeast cells.

## Data Availability

The data presented in this study are available upon request from the corresponding author.

## Supplementary Materials

The following supporting information can be downloaded at: https://www.mdpi.com/article/10.3390/antiox13050589/s1, Table S1: The strains and plasmids used in this study, Table S2: List of genes with significant changes in transcription levels, Figure S1: MS spectra of Cys_276_-containing peptide from YCA1, Figure S2: Flow cytometry analysis of BY4742 live and dead cells.
