# Supplementary material for: The Activity of YCA1 Metacaspase Is Regulated by Reactive Sulfane Sulfur via Persulfidation in Saccharomyces cerevisiae
Source: Antioxidants (Basel). 2024 May 10;13(5):589. doi: 10.3390/antiox13050589 (PMC11118234; doi:10.3390/antiox13050589)
Supplement: Supplementary file 1 [file antioxidants-13-00589-s001.zip › antioxidants-2969087-supplementary.pdf]

Table S1. The strains and plasmids used in this study.

Strain/plasmid	Relevant characteristic(s)	source
S. cerevisiae		
BY4742	*MATα his3Δ1 leu2Δ0 lys2Δ0 ura3Δ0*	Lab stock
BY4742 *Δcys3*	*Δcys3*::*loxp*	This study
BY4742 *Δcys3 Δyca1*	*Δcys3*::*loxp Δyca1*::*loxp*	
BY4742 *BIR1-GFP*	*BIR1-GFP(S65T)-HIS3*	This study
BY4742 *Δcys3 BIR1-GFP*	*Δcys3::loxp BIR1-GFP(S65T)-HIS3*	This study
E. coli		
DH5*α*	*supE44 ΔlacU169(Φ80dlacZΔM15) hsdR17 recA1 endA1 gyrA96 thi-1 relA1*	Lab stock
BL21(DE3)	*F-ompT hsdSB (rB-mB-) gal (λ1857 ind1 Sam7 nin5 lacUV5 T7gene1) dcm.*	Lab stock
**Plasmid**		
pUG6	Template plasmid	Lab stock
pFA6a-GFP(S65T)-His3MX6	Template plasmid	Lab stock
pET21b	Expression plasmid in *E. coli*	Lab stock
pET15b	Expression plasmid in *E. coli*	Lab stock
pET21b-*YCA1*	*YCA1* in pET21b, control by IPTG-induced lac promoter	This study
pET15b-*BIR1*	*BIR1* in pET15b, control by IPTG-induced lac promoter	This study

Table S2. List of gene with significant changes in transcription levels.

	gene	Log_2(Fold change)	p-value
	spl2	2.5752	1.72E-62
	pho84	2.5095	1.56 E-12
	ydr316w-b	2.473	0.0023443
	met17	2.3628	3.46 E-16
	met14	2.3466	2.55 E-10
	pho12	2.2886	2.86E-141
	ddr2	2.2731	6.47E-45
	met3	2.1144	4.20E-286
	ylr466c-b	2.0996	2.46E-20
	pho89	2.057	3.59E-94
	sul2	1.9295	9.64E-210
	tsl1	1.9015	1.36E-133
	dpi8	1.8684	3.06E-26
	met5	1.7585	4.25E-255
	yfl064c	1.7105	8.39E-06
	vtc3	1.6632	1.74E-287
	hsp82	1.6142	5.54E-22
	hsp42	1.5641	2.99E-53
	pho11	1.5456	1.14E-27
Up-regulated	*met10*	1.5018	5.01E-178
	pho5	1.4952	2.32E-106
	mht1	1.4498	2.87E-47
	yfl065c	1.4458	0.00063113
	str3	1.3663	3.57E-28
	ylr108c	1.3193	6.33E-67
	glk1	1.3106	1.69E-122
	gsy1	1.275	1.92E-42
	met16	1.2458	1.21E-44
	hsp12	1.2345	0.00013133
	yrf1-1	1.2283	1.59E-09
	hsp78	1.2188	9.10E-47
	yol162w	1.215	0.0029863
	phm6	1.2129	7.08E-16
	yll066c	1.2091	4.59E-44
	whi2	1.1887	7.20E-136
	mmp1	1.1862	1.81E-87
	mup1	1.182	3.47E-167
	ypr204w	1.1734	1.07E-18
	btn2	1.1629	1.93E-29
	cyc7	1.1489	1.85E-05
	hsp104	1.1481	4.13E-64

	opt1	1.1402	4.73E-127
Up-regulated	*yfl067w*	1.1321	1.20E-12
	hsp26	1.1245	1.19E-39
	hxk1	1.1108	9.82E-52
	yrf1-5	1.107	1.52E-11
	gad1	1.0961	4.80E-50
	cwp1	1.0955	5.26E-136
	gpm2	1.0708	4.44E-08
	pdc6	1.0708	0.0037978
	sfc1	1.0643	0.0011762
	cur1	1.0489	4.74E-14
	yol163w	1.0487	0.0012575
	seo1	1.0376	1.97E-05
	cit1	1.0268	2.15E-100
	met1	1.0125	1.47E-47
	yfl066c	1.0106	2.29E-08
	sam3	1.0062	6.86E-109
	tis11	1.0059	9.67E-14
Down-regulated	*snr189*	-1.0043	0.0026016
	mae1	-1.0343	6.90E-117
	aro5	-1.0689	6.64E-27
	arg4	-1.0777	1.12E-19
	ypr145c-a	-1.0788	2.18E-42
	ura1	-1.1201	2.37E-107
	pbi1	-1.1527	1.52E-129
	bsc1	-1.1614	1.43E-54
	prm7	-1.2831	4.82E-30
	snr35	-1.3268	9.56E-06
	cpa2	-1.5119	6.04E-22
	arg3	-1.5172	8.96E-25
	arg1	-1.553	1.62E-28
	srd1	-1.6645	9.09E-60
	tf(gaa)h1	-1.8982	0.010531
	pau9	-2.0243	0.00029412
	ybl107w-a	-3.6909	0.0035597
	tk(cuu)f	-4.2844	0.013111

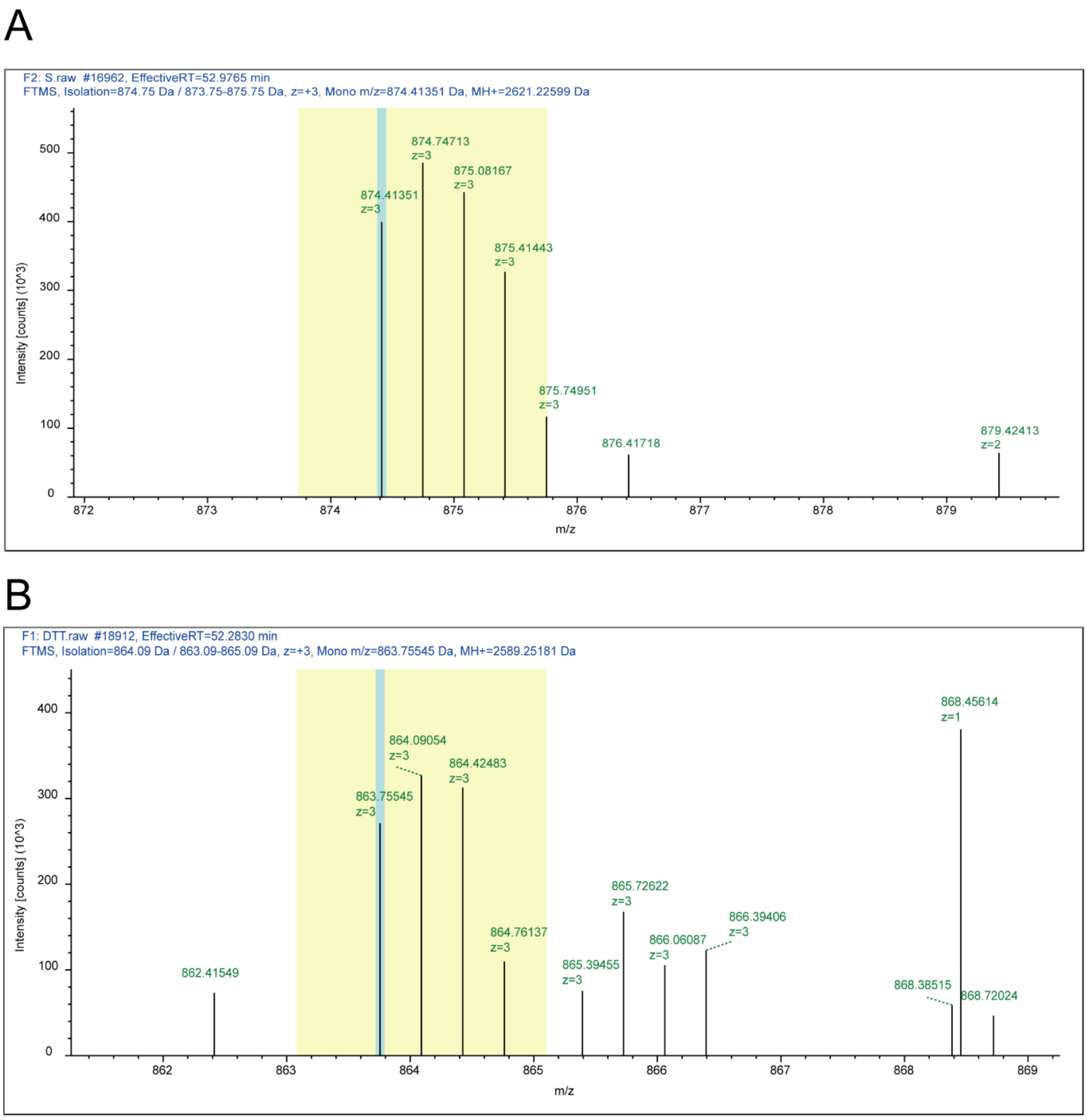

Figure S1. MS spectra of the Cys_{276} containing peptide from YCA1. (A) The peptide from HS_nH-reacted YCA1. (B) The peptide from DTT-reacted YCA1. "Z" represents the charge carried by the detected peptide segment. The molecular weights marked in blue indicate the detected values. The actual molecular weight is obtained by multiplying the detected molecular weight by the charge number.

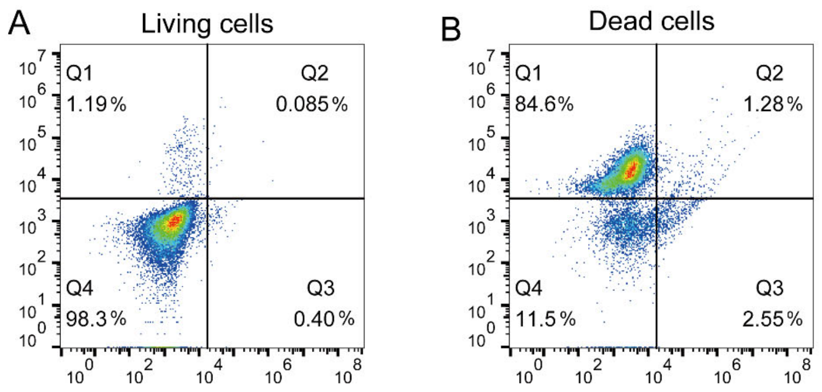

Figure S2. Flow cytometry analysis of BY4742 living and dead cells. A total of 20,000 stained cells were analyzed using flow cytometry to classify them into four quadrants. The cells underwent V-FITC and PI double staining. Viable cells (A) consisted of logarithmically growing yeast cells, while dead cells (B) were yeast cells killed by high temperature.
